# Effect of smoking status on clinical outcomes after reperfusion therapy for acute ischemic stroke

**DOI:** 10.1038/s41598-024-59508-3

**Published:** 2024-04-23

**Authors:** Fumi Irie, Ryu Matsuo, Satomi Mezuki, Yoshinobu Wakisaka, Masahiro Kamouchi, Takanari Kitazono, Tetsuro Ago, the Fukuoka Stroke Registry Investigators, Takao Ishitsuka, Setsuro Ibayashi, Kenji Kusuda, Kenichiro Fujii, Tetsuhiko Nagao, Yasushi Okada, Masahiro Yasaka, Hiroaki Ooboshi, Takanari Kitazono, Katsumi Irie, Tsuyoshi Omae, Kazunori Toyoda, Hiroshi Nakane, Masahiro Kamouchi, Hiroshi Sugimori, Shuji Arakawa, Kenji Fukuda, Tetsuro Ago, Jiro Kitayama, Shigeru Fujimoto, Shoji Arihiro, Junya Kuroda, Yoshinobu Wakisaka, Yoshihisa Fukushima, Ryu Matsuo, Fumi Irie, Kuniyuki Nakamura, Takuya Kiyohara

**Affiliations:** 1 Department of Health Care Administration and Management, Graduate School of Medical Sciences, Kyushu University, 3-1-1 Maidashi, Higashi-ku, Fukuoka, 812-8582 Japan; 2 Department of Medicine and Clinical Science, Graduate School of Medical Sciences, Kyushu University, 3-1-1 Maidashi, Higashi-ku, Fukuoka, Japan; 3 Center for Cohort Studies, Graduate School of Medical Sciences, Kyushu University, 3-1-1 Maidashi, Higashi-ku, Fukuoka, Japan; 4 Emergency and Clinical Care Center, Kyushu University Hospital, 3-1-1 Maidashi, Higashi-ku, Fukuoka, Japan; 5 Fukuoka Mirai Hospital, Fukuoka, Japan; 6 Seiai Rehabilitation Hospital, Onojo, Japan; 7 Japan Seafarers Relief Association Moji Ekisaikai Hospital, Kitakyushu, Japan; 8 Safety Monitoring Committee, Seiai Rehabilitation Hospital, Onojo, Japan; 9 National Hospital Organization Kyushu Medical Center, Fukuoka, Japan; 10 Fukuoka Neurosurgical Hospital, Fukuoka, Japan; 11 Fukuoka Dental College Medical and Dental Hospital, Fukuoka, Japan; 12 Kyushu University, Fukuoka, Japan; 13 Hakujyuji Hospital, Fukuoka, Japan; 14 Imazu Red Cross Hospital, Fukuoka, Japan; 15 National Cerebral and Cardiovascular Center, Suita, Japan; 16 National Hospital Organization Fukuoka-Higashi Medical Center, Koga, Japan; 17 Steel Memorial Yawata Hospital, Kitakyushu, Japan; 18 St Mary’s Hospital, Kurume, Japan; 19 Fukuoka Red Cross Hospital, Fukuoka, Japan; 20 Jichi Medical University, Shimotsuke, Japan; 21 Japan Labor Health and Welfare Organization Kyushu Rosai Hospital, Kitakyushu, Japan; 22 Kyushu University Hospital, Fukuoka, Japan

**Keywords:** Cerebrovascular disorders, Stroke

## Abstract

Smoking has detrimental effects on the cardiovascular system; however, some studies have reported better clinical outcomes after thrombolysis for ischemic stroke in smokers than in nonsmokers, a phenomenon known as the smoking paradox. Therefore, this study aimed to examine the smoking paradox in patients with ischemic stroke receiving reperfusion therapy. Data were collected from a multicenter hospital-based acute stroke registry in Fukuoka, Japan. The 1148 study patients were categorized into current and noncurrent smokers. The association between smoking and clinical outcomes, including neurological improvement (≥ 4-point decrease in the National Institutes of Health Stroke Scale during hospitalization or 0 points at discharge) and good functional outcomes (modified Rankin Scale score of 0–2) at 3 months, was evaluated using logistic regression analysis and propensity score-matched analysis. Among the participants, 231 (20.1%) were current smokers. The odds ratios (ORs) of favorable outcomes after adjusting for potential confounders were not significantly increased in current smokers (OR 0.85, 95% confidence interval [CI] 0.60–1.22 for neurological improvement; OR 0.95, 95% CI 0.65–1.38 for good functional outcome). No significant association was found in the propensity score-matched cohorts. Smoking cessation is strongly recommended since current smoking was not associated with better outcomes after reperfusion therapy.

## Introduction

Cigarette smoking has been proven harmful to the cardiovascular system^1–5^; however, some studies have suggested better outcomes following thrombolytic treatment in smokers than in nonsmokers. This phenomenon, which is known as the smoking-thrombolysis paradox, was first identified in patients with acute myocardial infarction^6–8^. Smokers appear to have better outcomes following thrombolysis than nonsmokers, possibly because of the increased susceptibility of thrombi in a hypercoagulable state induced by smoking to thrombolytic therapy^9–12^.

The smoking-thrombolysis paradox has also been examined in patients with acute ischemic stroke receiving intravenous thrombolysis. However, conflicting results have emerged, with some studies^13,14^ noting favorable effects of smoking on outcomes after thrombolytic therapy, while others have reported no effects^15–17^ or even negative effects^18^. Moreover, two studies examining the effects of smoking on clinical outcomes following reperfusion therapy, including intravenous thrombolysis and endovascular treatment, reached different conclusions. One study reported an association between current smoking and excellent clinical outcomes measured usingthe modified Rankin Scale (mRS) at 90 days poststroke^19^, while the other found no significant association between smoking and 3-month favorable functional outcomes^20^.

Residual confounding is one possible explanation for these conflicting results because smokers and nonsmokers have different background characteristics. Available evidence suggests that smokers are more likely to be younger and male and less frequently experience cardioembolic stroke than nonsmokers^14,18–21^. Some studies have also reported milder neurological deficits among smokers than among nonsmokers^19^. Age, sex, ischemic stroke subtype, and stroke severity can confound the association between smoking and poststroke outcomes since they are well-known factors that affect clinical outcomes after ischemic stroke^22,23^. Therefore, considering these differences in background characteristics between smokers and nonsmokers is important to properly examine the smoking-thrombolysis paradox in patients with ischemic stroke. However, most previous studies had relatively small cohort sizes, making thorough adjustments for confounders challenging.

Experimental studies have shown that smoking can have harmful effects on the cerebrovascular system, such as endothelial dysfunction and reduced cerebral blood flow^24,25^. Additionally, consistent with these basic findings, observational studies on the overall cohort of patients with ischemic stroke have reported poorer functional outcomes in smokers than in nonsmokers. Even if recanalization occurs more easily with fibrin-rich thrombi in smokers^26,27^, it may not necessarily lead to a better functional outcome in smokers because neurorestroration is hampered by smoking-induced vascular dysfunction^28,29^. From a clinical perspective, clarifying whether the possible benefit of successful clot dissolution in smokers results in true differences in poststroke clinical outcomes between smokers and nonsmokers is crucial.

Therefore, this study aimed to examine whether current smoking is associated with better clinical outcomes among patients with ischemic stroke receiving reperfusion therapy by carefully considering the differences in background characteristics between smokers and nonsmokers, using a large multicenter hospital-based stroke registry in Fukuoka, Japan.

## Results

### Background characteristics

The mean (standard deviation) age of the 1148 patients was 72.4 (12.2) years; 39.1% were female, and 231 (20.1%) were current smokers. Table 1 presents the background characteristics of current and noncurrent smokers. Current smokers were younger and comprised a larger proportion of males than noncurrent smokers. The frequencies of atrial fibrillation and chronic kidney disease and the frequency of alcohol consumption were lower and higher in current smokers, respectively than in noncurrent smokers. Additionally, current smokers had less frequent cardioembolism and presented lower National Institutes of Health Stroke Scale (NIHSS) scores on admission than noncurrent smokers.

**Table 1:** Background characteristics according to smoking status.

	Overall	Current smokers	Noncurrent smokers	*P*
	n = 1,148	n = 231	n = 917	
Age, year, mean ± SD	72.4 ± 12.2	65.2 ± 12.4	74.2 ± 11.5	< 0.001
Females, n (%)	449 (39.1)	36 (15.6)	413 (45.0)	< 0.001
Risk factors, n (%)				
Hypertension	869 (75.7)	174 (75.3)	695 (75.8)	0.88
Diabetes mellitus	251 (21.9)	57 (24.7)	194 (21.2)	0.25
Dyslipidemia	566 (49.3)	110 (47.6)	456 (49.7)	0.57
Atrial fibrillation	559 (48.7)	81 (35.1)	478 (52.1)	< 0.001
Alcohol consumption	466 (40.6)	146 (63.2)	320 (34.9)	< 0.001
Comorbidities, n (%)				
Coronary artery disease	165 (14.4)	35 (15.2)	130 (14.2)	0.71
Chronic kidney disease	503 (43.8)	76 (32.9)	427 (46.6)	< 0.001
Previous stroke, n (%)	141 (12.3)	21 (9.1)	120 (13.1)	0.10
Stroke subtype, n (%)				
Cardioembolism	577 (50.3)	85 (36.8)	492 (53.7)	< 0.001
Non-cardioembolism	571 (49.7)	146 (63.2)	425 (46.4)	
Large artery atherosclerosis	148 (12.9)	38 (16.5)	110 (12.0)	
Small vessel occlusion	117 (10.2)	36 (15.6)	81 (8.8)	
Others	306 (26.7)	72 (31.2)	234 (25.5)	
NIHSS score, median (IQR)	11 (5–18)	9 (5–16)	11 (6–18)	0.01
Reperfusion therapy, n (%)				
Intravenous thrombolysis	998 (86.9)	200 (86.6)	798 (87.0)	0.86
Endovascular therapy	346 (30.1)	81 (35.1)	265 (28.9)	0.07

SD: standard deviation, NIHSS: National Institutes of Health Stroke Scale, IQR: interquartile range.

### Smoking status and clinical outcomes

Table 2 shows the poststroke clinical outcomes according to smoking status. The frequency of neurological improvement was similar between current and noncurrent smokers. A multivariate-adjusted odds ratio of neurological improvement was not higher for current smokers than for noncurrent smokers. Additionally, the frequency of good functional outcomes in current smokers was higher than that in noncurrent smokers. Although the crude odds ratio of good functional outcomes was significantly higher in current smokers than in noncurrent smokers, this better outcome was not observed after adjusting for potential confounding factors. Among current smokers, we assessed the association between the number of cigarettes smoked daily and clinical outcomes (Table 3). Consequently, no association was found for current smokers who smoked < 20 cigarettes daily. Better functional outcome was observed among current smokers who smoked ≥ 20 cigarettes daily in a univariate analysis; however, this association was no longer found in multivariate analyses.

**Table 2:** Association between smoking status and clinical outcomes.

	Events, n (%)	Crude			Age and sex-adjusted			Multivariate-adjusted		
		OR	95% CI	*P*	OR	95% CI	*P*	OR	95% CI	*P*
Neurological improvement										
Noncurrent smokers, n = 917	651 (71.0)	1.00	(Reference)		1.00	(Reference)		1.00	(Reference)	
Current smokers, n = 231	160 (69.3)	0.92	(0.67–1.26)	0.61	0.88	(0.63–1.23)	0.45	0.85	(0.60–1.22)	0.39
Good functional outcome										
Noncurrent smokers, n = 917	515 (56.2)	1.00	(Reference)		1.00	(Reference)		1.00	(Reference)	
Current smokers, n = 231	157 (68.0)	1.66	(1.22–2.25)	0.001	1.03	(0.74–1.45)	0.84	0.95	(0.65–1.38)	0.79

OR: odds ratio, CI: confidence interval.

Neurological improvement was defined as a ≥ 4-point decrease in the National Institutes of Health Stroke Scale (NIHSS) score during hospitalization or 0 points at discharge. Good functional outcome was defined as a modified Rankin Scale score of 0–2 at 3 months after onset. The multivariate model included age, sex, hypertension, diabetes mellitus, dyslipidemia, atrial fibrillation, alcohol consumption, coronary artery disease, chronic kidney disease, previous stroke, stroke subtype, baseline NIHSS score, intravenous thrombolysis, and endovascular therapy.

**Table 3:** Association between quantified smoking status and clinical outcomes.

	Events, n (%)	Crude			Age and sex-adjusted			Multivariate-adjusted		
		OR	95% CI	*P*	OR	95% CI	*P*	OR	95% CI	*P*
Neurological improvement										
Noncurrent smokers, n = 917	651 (71.0)	1.00	(Reference)		1.00	(Reference)		1.00	(Reference)	
Current smokers (< 20), n = 79	57 (72.2)	1.06	(0.63–1.77)	0.83	1.03	(0.61–1.73)	0.91	1.01	(0.59–1.73)	0.98
Current smokers (≥ 20), n = 152	103 (67.8)	0.86	(0.59–1.24)	0.42	0.80	(0.54–1.19)	0.28	0.82	(0.54–1.24)	0.34
Good functional outcome										
Noncurrent smokers, n = 917	515 (56.2)	1.00	(Reference)		1.00	(Reference)		1.00	(Reference)	
Current smokers (< 20), n = 79	50 (63.3)	1.35	(0.84–2.17)	0.22	1.00	(0.61–1.65)	1.00	1.14	(0.66–1.97)	0.65
Current smokers (≥ 20), n = 152	107 (70.4)	1.86	(1.28–2.69)	0.001	1.06	(0.70–1.59)	0.80	0.92	(0.59–1.44)	0.73

OR: odds ratio, CI: confidence interval.

Current smokers were classified into two groups according to the number of cigarettes smoked per day (< 20 and ≥ 20).

Neurological improvement was defined as a ≥ 4-point decrease in the National Institutes of Health Stroke Scale (NIHSS) score during hospitalization or 0 points at discharge. Good functional outcome was defined as a modified Rankin Scale score of 0–2 at 3 months after onset. The multivariate model included age, sex, hypertension, diabetes mellitus, dyslipidemia, atrial fibrillation, alcohol consumption, coronary artery disease, chronic kidney disease, previous stroke, stroke subtype, baseline NIHSS score, intravenous thrombolysis, and endovascular therapy.

**Figure 1:**
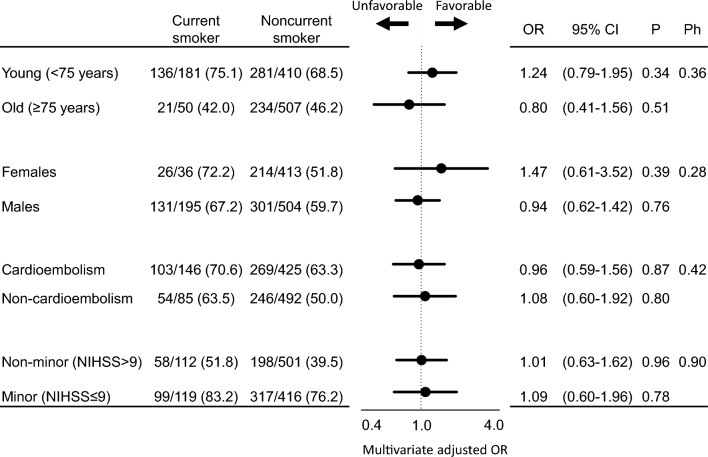
Association between current smoking and functional outcomes. OR: odds ratio, CI: confidence interval, Ph: *P*-value for heterogeneity. ORs and 95% CIs of good functional outcomes are shown according to the smoking status in each subgroup. The subgroups included age (< 75 or ≥ 75 years), sex, stroke subtype (cardioembolism or non-cardioembolism), and stroke severity (minor stroke or non-minor stroke). The multivariate model included age, sex, hypertension, diabetes mellitus, dyslipidemia, atrial fibrillation, alcohol consumption, coronary artery disease, chronic kidney disease, previous stroke, stroke subtype, baseline National Institutes of Health Stroke Scale (NIHSS) score, intravenous thrombolysis, and endovascular therapy. The Ph was evaluated by adding an interaction term to the multivariate model. Minor stroke was defined as an NIHSS score of ≤ 9 on admission.

Subgroup analyses of the association between smoking status and functional outcomes were performed according to age, sex, stroke subtype, and stroke severity to further assess whether specific populations are susceptible to the impact of smoking (Fig. 1). No significant differences were found in the association between smoking status and functional outcomes in any subgroup.

### Propensity score-matched analysis

This study evaluated the association between smoking status and clinical outcomes using propensity score-matched analysis to exclude possible bias caused by differences in background characteristics. No difference was found between current and noncurrent smokers for any variable in the propensity score-matched cohort (see Supplementary Table S1 online). The odds ratios of neurological improvement and good functional outcomes were not significantly higher in current smokers than in noncurrent smokers (Table 4).

**Table 4:** Association between smoking status and clinical outcomes in the propensity score-matched cohort.

	Events, n (%)	OR	95% CI	P*
Neurological improvement				
Noncurrent smokers, n = 201	140 (69.7)	1.00	(Reference)	
Current smokers, n = 201	140 (69.7)	1.00	(0.65–1.55)	1.00
Good functional outcome				
Noncurrent smokers, n = 201	128 (63.7)	1.00	(Reference)	
Current smokers, n = 201	132 (65.7)	1.10	(0.70–1.72)	0.75

OR: odds ratio, CI: confidence interval.

Neurological improvement was defined as a ≥ 4-point decrease in the National Institutes of Health Stroke Scale score during hospitalization or 0 points at discharge. Good functional outcome was defined as a modified Rankin Scale score of 0–2 at 3 months poststroke.

*P-values for the McNemar test.

### Sensitivity analysis

Similar differences in background characteristics, as noted when patients were categorized into two groups, were observed when they were classified into three groups according to smoking status (current, former, and never smokers) (see Supplementary Table S2 online). The multivariate-adjusted odds ratios of favorable outcomes in current smokers did not increase compared to that in never smokers (see Supplementary Table S3 online).

Analyses were also performed on a cohort of patients who received intravenous thrombolysis. Consequently, the crude odds ratio for good functional outcomes was higher in current smokers than in noncurrent smokers. However, smoking status was not associated with favorable outcomes after adjusting for possible confounding factors (see Supplementary Table S4 online). Similar results were obtained in the propensity score-matched cohort of patients who received intravenous thrombolysis as that of all study patients (see Supplementary Tables S4 and S5 online).

## Discussion

This study demonstrated no significant difference in neurological improvement during hospitalization between current and noncurrent smokers, either in univariate or multivariate analyses. The frequency of good functional outcomes at 3 months poststroke was higher among current smokers, particularly among those who smoked ≥ 20 cigarettes daily, than among noncurrent smokers. However, this difference was not observed after adjusting for possible confounders. No significant association was observed between smoking status and good functional outcomes in the propensity score-matched cohort. Furthermore, these results were essentially similar when the analysis was limited to patients receiving intravenous thrombolysis.

Some reports have suggested the beneficial effects of smoking on ischemic stroke outcomes among patients treated with intravenous thrombolysis or endovascular therapy^13,19,30^. However, since various factors determine poststroke functional outcomes^22,23^, the apparent association may be caused by the differences in clinical backgrounds between smokers and nonsmokers^21,31^. In this study, current smokers were younger, more frequently male, had a lower rate of atrial fibrillation and cardioembolism, and experienced less severe neurological deficits than noncurrent smokers. These characteristics are all known predictors of good functional outcomes after ischemic stroke^32–35^. Accordingly, this study found that the better functional outcomes observed in the crude analysis among current smokers than among noncurrent smokers were no longer found after controlling for background differences. No difference in functional outcomes was observed between smokers and noncurrent smokers in the propensity score-matched cohort. Furthermore, the subgroup analyses did not identify any group of patients where the influence of smoking status on functional outcomes was more pronounced. Therefore, these findings suggest that the apparent beneficial effects of smoking on post-reperfusion functional outcomes may be attributable to baseline differences between smokers and noncurrent smokers rather than smoking per se.

The existing literature indicates that smoking impairs the release of endogenous tissue plasminogen activator (tPA) and increases platelet activation, thereby causing higher intra-arterial fibrinogen and fibrin concentrations^11,12,36,37^. Additionally, the smoking-induced fibrin-rich clots may be more susceptible to fibrinolytic treatment^7,8^, possibly leading to improved tPA efficacy and a higher rate of recanalization in current smokers than in noncurrent smokers^14,38^. Consistent with this hypothesis, several studies have shown that smoking is associated with successful recanalization in patients with acute ischemic stroke treated with intravenous or intra-arterial tPA^20,21^. Theoretically, early recanalization can mitigate post-ischemic damage and lead to a higher rate of neurological improvement in smokers than in nonsmokers. However, this study showed no significant difference in neurological improvement during hospitalization between current and noncurrent smokers among patients receiving reperfusion therapy. A recent study using a large international database of patients treated with intravenous thrombolysis also reported a higher risk of early neurological deterioration in smokers than in nonsmokers^18^. These findings appear to contradict the idea that more efficient recanalization after thrombolysis results in better neurological recovery in current smokers than in noncurrent smokers. Therefore, further studies with detailed clinical information and imaging findings after reperfusion therapy are warranted to fully examine the theory of improved efficacy of thrombolytic treatment for ischemic stroke in smokers.

Furthermore, even if thrombi in current smokers are susceptible to fibrinolysis, smoking potentially damages endothelial cells, causes vascular dysfunction, and decreases cerebral blood flow^24,25^. These harmful effects of smoking may hamper poststroke neurorestoration through vascular remodeling and lead to poor functional recovery^28,29^. Previous studies, including ours, have suggested that functional outcomes were poorer in smokers than in nonsmokers among patients with ischemic stroke^26,27^. Our study, which includes patients with ischemic stroke receiving reperfusion therapy, found no significant association between smoking and functional outcomes at 3 months poststroke. Consistent with these observations, one study indicating a higher rate of recanalization in smokers reported no significant difference in 3-month functional outcomes between smokers and nonsmokers^21^. Moreover, another study reported poor functional recovery in smokers at 3 months poststroke after receiving intravenous thrombolysis^18^. These findings imply that the detrimental effects of smoking on the microvasculature in the brain might counterbalance or outweigh the possible beneficial influence of smoking on thrombolysis. A recent experimental study showed that reperfusion after ischemia enhances the survival of vascular cells, which contributes to efficient peri-infarct reorganization and better functional recovery^29^. However, further studies are needed to elucidate the pathophysiological effects of smoking on post-reperfusion repair processes involving vascular cells. Meanwhile, smoking cessation is strongly recommended, considering the harmful effects of smoking on the cerebrovascular system.

This study had some limitations. First, the possibility of misclassifying smoking status could not be excluded because the information was based on self-reported data. Second, the influence of passive exposure to cigarette smoke was not considered; therefore, passive smokers may have been included as nonsmokers. Third, the indication criteria and protocols for acute reperfusion therapy varied depending on the period and institution. The therapeutic window for intravenous thrombolysis had been within ≤ 3 h of stroke onset until 2012, after which it was extended to ≤ 4.5 h. Additionally, endovascular therapy was performed at the attending neurologist’s discretion at each participating hospital. Therefore, this heterogeneity in treatment might have hindered the analyses of the pure smoking-outcome relationship. Fourth, excluding patients with missing data from the analyses may have resulted in a selection bias. Finally, because all Fukuoka Stroke Registry (FSR) participating hospitals are tertiary care centers located in a restricted area of Japan, the generalizability of this study’s findings should be evaluated in other settings.

In conclusion, current smoking was not associated with neurological improvement during hospitalization or good functional outcomes at 3 months after acute ischemic stroke in patients treated with reperfusion therapy. Therefore, considering the detrimental effects of smoking on vascular function and neurological recovery, smoking cessation is strongly recommended. However, further studies are needed to improve the mechanistic understanding of the effects of smoking on the repair processes in ischemic areas after reperfusion.

## Methods

### Study design

This study included patients with stroke registered in the FSR, a multicenter, hospital-based registry of acute stroke (Appendix in Supplementary Information)^39,40^. The registry enrolled patients with stroke who were admitted to seven participating hospitals within 7 days of stroke onset (University Hospital Medical Information Clinical Trial Registry: UMIN-CTR, Unique ID: UMIN000000800, 2007/9/1). This study was performed in accordance with the principle of the Declaration of Helsinki, and the Institutional Review Boards of the following participating hospitals approved its design: Kyushu University Institutional Review Board for Clinical Research, 22086-00; Kyushu Medical Center Institutional Review Board, R06-03; Clinical Research Review Board of Fukuoka-Higashi Medical Center, 29-C-38; Fukuoka Red Cross Hospital Institutional Review Board, 629; St. Mary’s Hospital Research Ethics Review Committee, S13-0110; Steel Memorial Yawata Hospital Ethics Committee, 06-04-13; and Kyushu Rosai Hospital Institutional Review Board, 21-8. Written informed consent was obtained from all the participants, and permission was obtained from patients’ family members if they could not provide consent.

Stroke was defined as the sudden onset of nonconvulsive and focal neurological deficits. Ischemic stroke was diagnosed using brain computed tomography, magnetic resonance imaging, or both. Reperfusion therapy included intravenous thrombolysis using recombinant tPA and endovascular therapies, such as mechanical thrombectomy and intra-arterial thrombolysis.

### Patient selection

A total of 14,501 patients with acute ischemic stroke were registered in the FSR between July 2007 and November 2018. Of these, 13,005 patients who did not receive acute reperfusion therapy were excluded. Among the 1,496 patients who received acute reperfusion therapy, 321 with impaired activities of daily living before stroke onset that was defined with a mRS score of ≥ 2, and 27 who could not be followed up at 3 months poststroke were excluded. Finally, this study analyzed the data of 1,148 patients with acute ischemic stroke who were independent before stroke onset and received acute reperfusion therapy (see Supplementary Fig. S1 online).

### Smoking status

On admission or during hospitalization, patients with stroke or their family members were asked about the patients’ pre-stroke smoking status using a questionnaire developed in Specific Health Checkups and Guidance in Japan^41^. The patients were categorized into two groups based on their smoking status: (i) current and (ii) noncurrent smokers. A current smoker was defined as a patient with a smoking history within 6 months preceding the index stroke. A noncurrent smoker was defined as a patient who had previously smoked (former smoker) but had stopped smoking for 6 months before the index stroke or a patient who had never smoked (never smoker).^5,19,42^ Current smokers were further categorized into two groups based on the number of cigarettes smoked per day: < 20 and ≥ 20^27^.

### Clinical assessment

The items to be examined as background characteristics were selected by considering their clinical relevance to poststroke outcomes and previous findings on the differences between smokers and nonsmokers. These items included age, sex, cardiovascular risk factors, comorbidities, history of any stroke, ischemic stroke etiology, and NIHSS score on admission. Cardiovascular risk factors and comorbidities were assessed based on previously described definitions^39,40^, whereas a previous stroke was defined as a history of hemorrhagic or ischemic stroke. Ischemic stroke was classified into four subtypes according to the Trial of ORG 10172 in Acute Stroke Treatment criteria as follows^43^: cardioembolism, large artery atherosclerosis, small vessel occlusion, and others. Stroke severity was assessed using the NIHSS score on admission, with minor stroke defined as an NIHSS score ≤ 9 on admission. Functional outcomes were assessed using the mRS score. Trained stroke neurologists assessed the NIHSS and mRS scores during hospitalization. Trained and certified research nurses evaluated the mRS score at 3 months poststroke through telephone assessment using a standardized structured questionnaire validated in a previous study aimed at minimizing the inter-rater variability^44^. We also collected information on acute reperfusion therapy during hospitalization, including thrombolytic therapy with intravenous recombinant tPA and endovascular therapy with intra-arterial thrombolysis, endovascular thrombectomy, thromboaspiration, or angioplasty.

### Study outcomes

The study outcomes were neurological improvement during hospitalization and good functional outcomes at 3 months after stroke onset. Neurological improvement was defined as a ≥ 4-point decrease in the NIHSS score during hospitalization or 0 points at discharge^45^. A good functional outcome was defined as an mRS score of 0–2 at 3 months poststroke^20,21^.

### Statistical analysis

Baseline characteristics according to smoking status were compared using the chi-square test, unpaired t-test, or Wilcoxon rank sum test, as appropriate. Logistic regression analysis was used to estimate the study outcomes’ multivariate-adjusted odds ratios and 95% confidence intervals. The multivariate model included age and the baseline NIHSS score as continuous variables and sex, hypertension, diabetes mellitus, dyslipidemia, atrial fibrillation, alcohol consumption, coronary artery disease, chronic kidney disease, previous stroke, stroke subtype, intravenous thrombolysis, and endovascular therapy as categorical variables. In the subgroup analyses, patients were categorized into two subgroups according to age (< 75 or ≥ 75 years), sex, stroke subtype (cardioembolism or non-cardioembolism), and stroke severity (minor stroke or non-minor stroke). P-values for heterogeneity were calculated by adding the interaction term of smoking status and the variable of interest to the model.

Propensity score-matched analysis was performed to rule out selection bias by controlling baseline differences between current and noncurrent smokers. Regarding propensity score matching, logistic regression modeling was used to calculate propensity scores incorporating the following variables: age, sex, hypertension, diabetes mellitus, dyslipidemia, atrial fibrillation, alcohol consumption, coronary artery disease, chronic kidney disease, previous stroke, stroke subtype, NIHSS score on admission, intravenous thrombolysis, and endovascular therapy. Patients with and without current smoking underwent one-to-one nearest neighbor (greedy type) matching of the standard deviation of the propensity score logit with a caliper width of 0.25. Matching was performed without replacement, and unpaired cases and controls that did not meet the matching criteria were excluded. Each propensity score-derived matched pair was assigned a unique pair identification number, and 201 matched-pair identification numbers were selected.

The sensitivity analyses examined the associations after categorizing smoking status into three groups: current, former, and never smokers. This study also evaluated the association between current smoking status and clinical outcomes after restricting the analysis to patients treated with intravenous thrombolysis.

Statistical analyses were performed using Stata 15 software (StataCorp LP, College Station, TX, USA), and a two-tailed *P*-value < 0.05 was considered statistically significant.

### Supplementary Information

Supplementary Information.

## Data Availability

An anonymized copy of the data used in this study can be obtained from the corresponding author upon reasonable request from a qualified researcher with the permission of the local institutional review board.

## References

[1] Abbott RD, Yin Y, Reed DM, Yano K (1986). Risk of stroke in male cigarette smokers. N. Engl. J. Med..

[2] Shinton R, Beevers G (1989). Meta-analysis of relation between cigarette smoking and stroke. BMJ.

[3] Thun MJ (2013). 50-year trends in smoking-related mortality in the United States. N. Engl. J. Med..

[4] Bhat VM (2008). Dose-response relationship between cigarette smoking and risk of ischemic stroke in young women. Stroke.

[5] Mannami T (2004). Cigarette smoking and risk of stroke and its subtypes among middle-aged Japanese men and women: The JPHC Study Cohort I. Stroke.

[6] Barbash GI (1993). Significance of smoking in patients receiving thrombolytic therapy for acute myocardial infarction. Experience leaned from the International Tissue Plasminogen Activator/Streptokinase Mortality Trial. Circulation.

[7] Gomez MA, Karagounis LA, Allen A, Anderson JL (1993). Effect of cigarette smoking on coronary patency after thrombolytic therapy for myocardial infarction. TEAM-2 Investigators. Second Multicenter Thrombolytic Trials of Eminase in Acute Myocardial Infarction. Am. J. Cardiol..

[8] Grines CL (1995). Effect of cigarette smoking on outcome after thrombolytic therapy for myocardial infarction. Circulation.

[9] Purcell IF, Newall N, Farrer M (1999). Lower cardiac mortality in smokers following thrombolysis for acute myocardial infarction may be related to more effective fibrinolysis. QJM.

[10] Zidovetzki R, Chen P, Fisher M, Hofman FM, Faraci FM (1999). Nicotine increases plasminogen activator inhibitor-1 production by human brain endothelial cells via protein kinase C-associated pathway. Stroke.

[11] Newby DE (2001). Impaired coronary tissue plasminogen activator release is associated with coronary atherosclerosis and cigarette smoking: Direct link between endothelial dysfunction and atherothrombosis. Circulation.

[12] Barua RS (2010). Effects of cigarette smoke exposure on clot dynamics and fibrin structure: An ex vivo investigation. Arterioscler. Thromb. Vasc. Biol..

[13] Ovbiagele B, Saver JL (2005). The smoking-thrombolysis paradox and acute ischemic stroke. Neurology.

[14] Tong X (2016). Smoking-thrombolysis relationship depends on ischemic stroke subtype. Stroke.

[15] Schlemm L (2019). Current smoking does not modify the treatment effect of intravenous thrombolysis in acute ischemic stroke patients-a post-hoc analysis of the WAKE-UP Trial. Front. Neurol..

[16] Zhang P, Guo ZN, Sun X, Zhao Y, Yang Y (2019). Meta-analysis of the smoker's paradox in Acute Ischemic Stroke Patients Receiving Intravenous Thrombolysis or Endovascular Treatment. Nicotine Tob. Res..

[17] Kufner A, Ebinger M, Luijckx GJ, Endres M, Siegerink B (2020). Smoking does not alter treatment effect of intravenous thrombolysis in mild to moderate acute ischemic stroke-a Dutch String-of-Pearls Institute (PSI) Stroke Study. Front. Neurol..

[18] Sun L (2021). Smoking influences outcome in patients who had thrombolysed ischaemic stroke: The ENCHANTED study. Stroke Vasc. Neurol..

[19] von Martial R (2018). Impact of smoking on stroke outcome after endovascular treatment. PLoS One.

[20] Meseguer E (2014). The smoking paradox: Impact of smoking on recanalization in the setting of intra-arterial thrombolysis. Cerebrovasc. Dis. Extra.

[21] Kufner A (2013). Smoking-thrombolysis paradox: Recanalization and reperfusion rates after intravenous tissue plasminogen activator in smokers with ischemic stroke. Stroke.

[22] Saposnik G (2011). The iScore predicts poor functional outcomes early after hospitalization for an acute ischemic stroke. Stroke.

[23] Ntaios G (2012). An integer-based score to predict functional outcome in acute ischemic stroke: The ASTRAL score. Neurology.

[24] Rogers RL (1983). Cigarette smoking decreases cerebral blood flow suggesting increased risk for stroke. JAMA.

[25] Messner B, Bernhard D (2014). Smoking and cardiovascular disease: Mechanisms of endothelial dysfunction and early atherogenesis. Arterioscler. Thromb. Vasc. Biol.

[26] Ovbiagele B (2006). Effect of smoking status on outcome after acute ischemic stroke. Cerebrovasc. Dis..

[27] Matsuo R (2020). Smoking status and functional outcomes after acute ischemic stroke. Stroke.

[28] Chen J, Venkat P, Zacharek A, Chopp M (2014). Neurorestorative therapy for stroke. Front. Hum. Neurosci..

[29] Tachibana M (2017). Early reperfusion after brain ischemia has beneficial effects beyond rescuing neurons. Stroke.

[30] Kvistad CE (2014). Is smoking associated with favourable outcome in tPA-treated stroke patients?. Acta Neurol. Scand..

[31] Aries MJ (2009). Does smoking influence outcome after intravenous thrombolysis for acute ischaemic stroke?. Eur. J. Neurol..

[32] Di Carlo A (2003). Sex differences in the clinical presentation, resource use, and 3-month outcome of acute stroke in Europe: Data from a multicenter multinational hospital-based registry. Stroke.

[33] Palnum KD (2008). Older patients with acute stroke in Denmark: quality of care and short-term mortality. A nationwide follow-up study. Age Ageing.

[34] Smith EE (2010). Risk score for in-hospital ischemic stroke mortality derived and validated within the Get With the Guidelines-Stroke Program. Circulation.

[35] Irie F (2015). Sex differences in short-term outcomes after acute ischemic stroke: The Fukuoka Stroke Registry. Stroke.

[36] Meade TW, Imeson J, Stirling Y (1987). Effects of changes in smoking and other characteristics on clotting factors and the risk of ischaemic heart disease. Lancet.

[37] Sambola A (2003). Role of risk factors in the modulation of tissue factor activity and blood thrombogenicity. Circulation.

[38] Tandberg Askevold E, Naess H, Thomassen L (2007). Predictors for recanalization after intravenous thrombolysis in acute ischemic stroke. J. Stroke Cerebrovasc. Dis..

[39] Kamouchi M (2011). Prestroke glycemic control is associated with the functional outcome in acute ischemic stroke: The Fukuoka Stroke Registry. Stroke.

[40] Kumai Y (2012). Proteinuria and clinical outcomes after ischemic stroke. Neurology.

[41] Tsushita K (2018). Rationale and descriptive analysis of specific health guidance: the nationwide lifestyle intervention program targeting metabolic syndrome in Japan. J. Atheroscler. Thromb..

[42] Tse LA, Fang XH, Wang WZ, Qiu H, Yu IT (2012). Incidence of ischaemic and haemorrhagic stroke and the association with smoking and smoking cessation: A 10-year multicentre prospective study in China. Public Health.

[43] Adams HP (1993). Classification of subtype of acute ischemic stroke. Definitions for use in a multicenter clinical trial. TOAST. Trial of Org 10172 in Acute Stroke Treatment. Stroke.

[44] Shinohara Y, Yamaguchi T (2008). Outline of the Japanese guidelines for the management of stroke 2004 and subsequent revision. Int. J. Stroke.

[45] Yong M, Kaste M (2008). Dynamic of hyperglycemia as a predictor of stroke outcome in the ECASS-II trial. Stroke.

