# Supplementary material for: Effect of smoking status on clinical outcomes after reperfusion therapy for acute ischemic stroke
Source: Sci Rep. 2024 Apr 23;14:9290. doi: 10.1038/s41598-024-59508-3 (PMC11039615; doi:10.1038/s41598-024-59508-3)
Supplement: Supplementary file 1 — Supplementary Information. [file 41598_2024_59508_MOESM1_ESM.pdf]

Supplementary Information

Effect of smoking status on clinical outcomes after reperfusion therapy for acute ischemic stroke

Fumi Irie, MD, PhD[1,2,3]; Ryu Matsuo, MD, PhD[1,2,3*]; Satomi Mezuki, MD[2,4]; Yoshinobu Wakisaka, MD, PhD[2]; Masahiro Kamouchi, MD, PhD[1,3]; Takanari Kitazono, MD, PhD[2,3]; Tetsuro Ago, MD, PhD[2], on behalf of the Fukuoka Stroke Registry Investigators.

[1]Department of Health Care Administration and Management, Graduate School of Medical Sciences, Kyushu University, 3-1-1 Maidashi, Higashi-ku, Fukuoka, Japan
[2]Department of Medicine and Clinical Science, Graduate School of Medical Sciences, Kyushu University, 3-1-1 Maidashi, Higashi-ku, Fukuoka, Japan
[3]Center for Cohort Studies, Graduate School of Medical Sciences, Kyushu University, 3-1-1 Maidashi, Higashi-ku, Fukuoka, Japan
[4]Emergency and Clinical Care Center, Kyushu University Hospital, 3-1-1 Maidashi, Higashi-ku, Fukuoka, Japan

Supplementary Tables

Table S1. Background characteristics according to smoking status in the propensity score-matched cohort

Table S2. Background characteristics according to the three-group smoking status

Table S3. Association between the three-group smoking status and clinical outcomes

Table S4. Association between smoking status and clinical outcomes in patients receiving intravenous thrombolysis

Table S5. Background characteristics according to the smoking status of patients receiving intravenous thrombolysis after propensity score matching

Supplementary Figure

Figure S1. Flow chart of patient selection

Appendix

Table S1. Background characteristics according to smoking status in the propensity score-matched cohort

	Current smokers n=201	Noncurrent smokers n=201	P
Age, year, mean ± SD	67.3±11.1	68.2±11.9	0.45
Females, n (%)	36 (17.9)	28 (13.9)	0.28
Risk factors, n (%)			
Hypertension	155 (77.1)	154 (76.6)	0.91
Diabetes mellitus	49 (24.4)	51 (25.4)	0.82
Dyslipidemia	93 (46.3)	98 (48.8)	0.62
Atrial fibrillation	77 (38.3)	74 (36.8)	0.76
Alcohol consumption	119 (59.2)	124 (61.7)	0.61
Comorbidities, n (%)			
Coronary artery disease	30 (14.9)	31 (15.4)	0.89
Chronic kidney disease	74 (36.8)	84 (41.8)	0.31
Previous stroke, n (%)	20 (10.0)	23 (11.4)	0.63
Stroke subtype, n (%)			0.76
Cardioembolism	82 (40.8)	79 (39.3)	
Non-cardioembolism	119 (59.2)	122 (60.7)	
Large artery atherosclerosis	58 (28.9)	64 (31.8)	
Small vessel occlusion	33 (16.4)	33 (16.4)	
Others	58 (28.9)	64 (31.8)	
NIHSS score, median (IQR)	10 (5–16)	9 (4–16)	0.78
Reperfusion therapy, n (%)			
Intravenous thrombolysis	174 (86.6)	175 (87.1)	0.88
Endovascular therapy	66 (32.8)	66 (32.8)	1.00

SD: standard deviation, NIHSS: National Institutes of Health Stroke Scale, IQR: interquartile range.

Table S2. Background characteristics according to the three-group smoking status

	Current smokers n=231	Former smokers n=388	Never smokers n=529	P
Age, year, mean ± SD	65.2±12.4	73.6±9.9	74.7±12.5	<0.001
Females, n (%)	36 (15.6)	43 (11.1)	370 (69.9)	<0.001
Risk factors, n (%)				
Hypertension	174 (75.3)	313 (80.7)	382 (72.2)	0.01
Diabetes mellitus	57 (24.7)	97 (25.0)	97 (18.3)	0.03
Dyslipidemia	110 (47.6)	192 (49.5)	264 (49.9)	0.84
Atrial fibrillation	81 (35.1)	196 (50.5)	282 (53.3)	<0.001
Alcohol consumption	146 (63.2)	211 (54.4)	109 (20.6)	<0.001
Comorbidities, n (%)				
Coronary artery disease	35 (15.2)	71 (18.3)	59 (11.2)	0.009
Chronic kidney disease	76 (32.9)	181 (46.7)	246 (46.5)	0.001
Previous stroke, n (%)	21 (9.1)	61 (15.7)	59 (11.2)	0.03
Stroke subtype, n (%)				<0.001
Cardioembolism	85 (36.8)	197 (50.8)	295 (55.8)	
Non-cardioembolism	146 (63.2)	191 (49.2)	234 (44.2)	
Large artery atherosclerosis	38 (16.5)	64 (16.5)	46 (8.7)	
Small vessel occlusion	36 (15.6)	33 (8.5)	48 (9.1)	
Others	72 (31.2)	94 (24.2)	140 (26.5)	
NIHSS score, median (IQR)	9 (5–16)	10 (5–18)	12 (6–18)	0.003
Reperfusion therapy, n (%)				
Intravenous thrombolysis	200 (86.6)	351 (90.5)	447 (84.5)	0.03
Endovascular therapy	81 (35.1)	102 (26.3)	163 (30.8)	0.06

SD: standard deviation, NIHSS: National Institutes of Health Stroke Scale, IQR: interquartile range.

Table S3. Association between the three-group smoking status and clinical outcomes

		Crude			Age and sex-adjusted			Multivariate-adjusted		
	Events, n (%)	OR	95% CI	P	OR	95% CI	P	OR	95% CI	P
Neurological improvement										
Never smokers, n=529	381 (72.0)	1.00	(reference)		1.00	(reference)		1.00	(reference)	
Former smokers, n=388	270 (69.6)	0.89	(0.67–1.19)	0.42	0.90	(0.64–1.27)	0.55	1.00	(0.69–1.44)	0.99
Current smokers, n=231	160 (69.3)	0.88	(0.62–1.23)	0.44	0.83	(0.56–1.22)	0.34	0.88	(0.58–1.33)	0.54
Good functional outcome										
Never smokers, n=529	280 (52.9)	1.00	(reference)		1.00	(reference)		1.00	(reference)	
Former smokers, n=388	235 (60.6)	1.37	(1.05–1.78)	0.02	1.28	(0.92–1.77)	0.15	1.22	(0.85–1.76)	0.28
Current smokers, n=231	157 (68.0)	1.89	(1.36–2.61)	<0.001	1.19	(0.81–1.76)	0.37	1.13	(0.73–1.73)	0.59

OR: odds ratio, CI: confidence interval
Neurological improvement was defined as a ≥4-point decrease in the National Institutes of Health Stroke Scale (NIHSS) score during hospitalization or 0 points at discharge. Good functional outcome was defined as a modified Rankin Scale score of 0–2 at 3 months poststroke. The multivariate model included age, sex, hypertension, diabetes mellitus, dyslipidemia, atrial fibrillation, alcohol consumption, coronary artery disease, chronic kidney disease, previous stroke, stroke subtype, baseline NIHSS score, intravenous thrombolysis, and endovascular therapy.

Table S4. Association between smoking status and clinical outcomes in patients receiving intravenous thrombolysis

		Crude			Age and sex-adjusted			Multivariate-adjusted		
	Events, n (%)	OR	95% CI	P	OR	95% CI	P	OR	95% CI	P
Main cohort										
Neurological improvement										
Noncurrent smokers, n=798	564 (70.7)	1.00	(reference)		1.00	(reference)		1.00	(reference)	
Current smokers, n=200	135 (67.5)	0.88	(0.62–1.23)	0.44	0.83	(0.56–1.22)	0.34	0.80	(0.55–1.16)	0.24
Good functional outcome										
Noncurrent smokers, n=798	463 (58.0)	1.00	(reference)		1.00	(reference)		1.00	(reference)	
Current smokers, n=200	135 (67.5)	1.37	(1.05–1.78)	0.02	1.28	(0.92–1.77)	0.15	0.83	(0.55–1.25)	0.37
Propensity score-matched cohort										
Neurological improvement		1.00	(reference)							
Noncurrent smokers, n=161	105 (65.2)			0.61*						
Current smokers, n=161	110 (68.3)									
Good functional outcome		1.00	(reference)							
Noncurrent smokers, n=161	108 (67.1)			0.65*						
Current smokers, n=161	103 (64.0)									

OR: odds ratio, CI: confidence interval

Neurological improvement was defined as a ≥4-point decrease in the National Institutes of Health Stroke Scale (NIHSS) score during hospitalization or 0 points at discharge. Good functional outcome was defined as a modified Rankin Scale score of 0–2 at 3 months poststroke. The multivariate model included age, sex, hypertension, diabetes mellitus, dyslipidemia, atrial fibrillation, alcohol consumption, coronary artery disease, chronic kidney disease, previous stroke, stroke subtype, and baseline NIHSS score.

* P-values for the McNemar test

Table S5. Background characteristics according to the smoking status of patients receiving intravenous thrombolysis after propensity score matching

	Current smokers n=161	Noncurrent smokers n=161	P
Age, year, mean ± SD	68.5±10.2	68.3±12.1	0.92
Females, n (%)	31 (19.3)	27 (16.8)	0.56
Risk factors, n (%)			
Hypertension	126 (78.3)	120 (74.5)	0.43
Diabetes mellitus	42 (26.1)	38 (23.6)	0.61
Dyslipidemia	75 (46.6)	68 (42.2)	0.43
Atrial fibrillation	63 (39.1)	70 (43.5)	0.43
Alcohol consumption	91 (56.5)	97 (60.3)	0.50
Comorbidities, n (%)			
Coronary artery disease	26 (16.2)	26 (16.2)	1.00
Chronic kidney disease	63 (39.1)	58 (36.0)	0.57
Previous stroke, n (%)	17 (10.6)	21 (13.0)	0.49
Stroke subtype, n (%)			0.37
Cardioembolism	65 (40.4)	73 (45.3)	
Non-cardioembolism	96 (59.6)	88 (54.7)	
Large artery atherosclerosis	20 (12.4)	22 (13.7)	
Small vessel occlusion	27 (16.8)	19 (11.8)	
Others	49 (30.4)	47 (29.2)	
NIHSS score, median (IQR)	9 (5–15)	8 (4–15)	
Reperfusion therapy, n (%)			
Intravenous thrombolysis	–	–	
Endovascular therapy	34 (21.1)	32 (19.9)	0.78

SD: standard deviation, NIHSS: National Institutes of Health Stroke Scale, IQR: interquartile range

Figure S1. Flow chart of patient selection

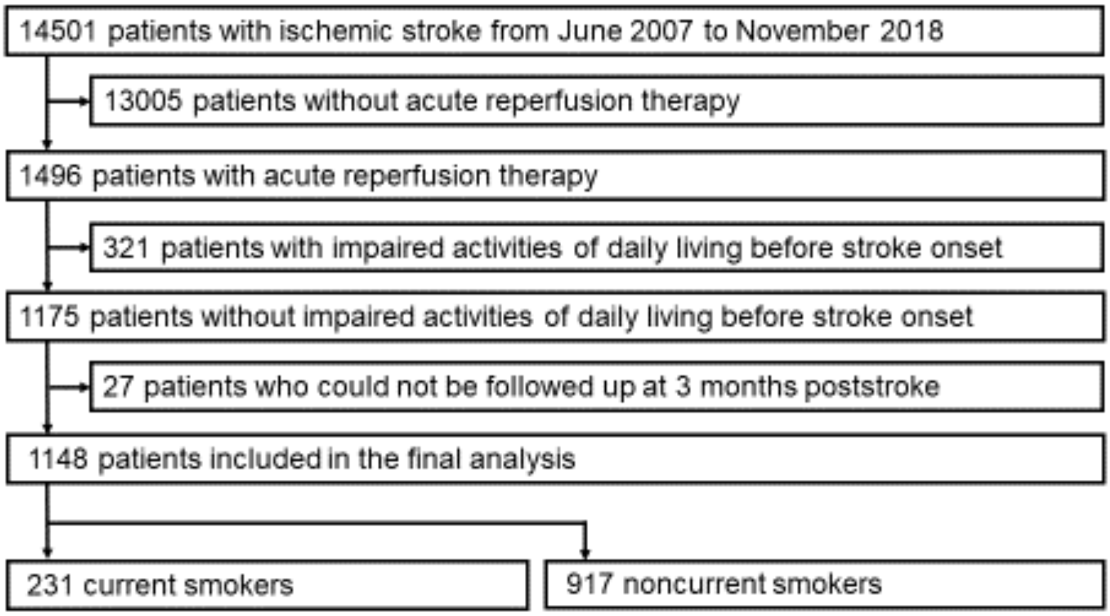

Appendix

Participating hospitals of the FSR

Kyushu University Hospital (Fukuoka, Japan), National Hospital Organization Kyushu Medical Center (Fukuoka, Japan), National Hospital Organization Fukuoka-Higashi Medical Center (Koga, Japan), Fukuoka Red Cross Hospital (Fukuoka, Japan), St Mary's Hospital (Kurume, Japan), Steel Memorial Yawata Hospital (Kitakyushu, Japan), and Japan Labor Health and Welfare Organization Kyushu Rosai Hospital (Kitakyushu, Japan).

Steering committee and research working group members of the FSR

Takao Ishitsuka, MD, PhD (Fukuoka Mirai Hospital, Fukuoka, Japan); Setsuro Ibayashi, MD, PhD (Chair, Seiai Rehabilitation Hospital, Onojo, Japan); Kenji Kusuda, MD, PhD (Seiai Rehabilitation Hospital, Onojo, Japan); Kenichiro Fujii, MD, PhD (Japan Seafarers Relief Association Moji Ekisaikai Hospital, Kitakyushu, Japan); Tetsuhiko Nagao, MD, PhD (Safety Monitoring Committee, Seiai Rehabilitation Hospital, Onojo, Japan); Yasushi Okada, MD, PhD (Vice-Chair, National Hospital Organization Kyushu Medical Center, Fukuoka, Japan); Masahiro Yasaka, MD, PhD (Fukuoka Neurosurgical Hospital, Fukuoka, Japan); Hiroaki Ooboshi, MD, PhD (Fukuoka Dental College Medical and Dental Hospital, Fukuoka, Japan), Takanari Kitazono, MD, PhD (Principal Investigator, Kyushu University, Fukuoka, Japan); Katsumi Irie, MD, PhD (Hakujyuji Hospital, Fukuoka, Japan); Tsuyoshi Omae, MD, PhD (Imazu Red Cross Hospital, Fukuoka, Japan); Kazunori Toyoda, MD, PhD (National Cerebral and Cardiovascular Center, Suita, Japan); Hiroshi Nakane, MD, PhD (National Hospital Organization Fukuoka-Higashi Medical Center, Koga, Japan); Masahiro Kamouchi, MD, PhD (Kyushu University, Fukuoka, Japan); Hiroshi Sugimori, MD, PhD (National Hospital Organization Kyushu Medical Center, Fukuoka, Japan); Shuji Arakawa, MD, PhD (Steel Memorial Yawata Hospital, Kitakyushu, Japan); Kenji Fukuda, MD, PhD (St Mary's Hospital, Kurume, Japan); Tetsuro Ago, MD, PhD (Kyushu University, Fukuoka, Japan); Jiro Kitayama, MD, PhD (Fukuoka Red Cross Hospital, Fukuoka, Japan); Shigeru Fujimoto, MD, PhD (Jichi Medical University, Shimotsuke, Japan), Shoji Arihiro, MD (Japan Labor Health and Welfare Organization Kyushu Rosai Hospital, Kitakyushu, Japan); Junya Kuroda, MD, PhD (National Hospital Organization Fukuoka-Higashi Medical Center, Koga, Japan); Yoshinobu Wakisaka, MD, PhD (Kyushu University Hospital, Fukuoka, Japan), Yoshihisa Fukushima, MD (St Mary's Hospital, Kurume, Japan); Ryu Matsuo, MD, PhD (Secretariat, Kyushu University, Fukuoka, Japan); Fumi Irie, MD, PhD (Kyushu University, Fukuoka, Japan); Kuniyuki Nakamura, MD, PhD (Kyushu University Hospital, Fukuoka, Japan); and Takuya Kiyohara, MD, PhD (Kyushu University Hospital, Fukuoka, Japan).
